# A STUDY ON THE ETIOLOGICAL AGENT AND CLINICO-MYCOLOGICAL CORRELATION OF FINGERNAIL ONYCHOMYCOSIS IN EASTERN INDIA

**DOI:** 10.4103/0019-5154.41651

**Published:** 2008

**Authors:** Nilay Kanti Das, Pramit Ghosh, Suchibrata Das, Susmita Bhattacharya, Rathindra Nath Dutta, Sujit Ranjan Sengupta

**Affiliations:** *From the Department of Dermatology, Medical College and Hospital, 88 College Street, Kolkata - 73, India*; 1 *From the Department of Community Medicine, Medical College and Hospital, 88 College Street, Kolkata - 73, India*; 2 *From the Department of Dermatology, Institute of Postgraduate Medical Education and Research, 244 AJC Bose Road, Kolkata - 20, India*; 3 *From the Department of Microbiology, NRS Medical College and Hospital, 138 AJC Bose Road, Kolkata - 14, India*

**Keywords:** *Etiological agents*, *nail changes*, *onychomycosis*

## Abstract

**Background:**

Onychomycosis manifests itself in various forms, notably onychodystrophy, onycholysis, subungual hyperkeratosis, or nail-plate discoloration. Not necessarily nail changes mentioned here should always be of fungal origin.

**Objective:**

The present study is planned to get an idea about etiological agent and clinical correlation in fingernail onychomycosis.

**Materials and Methods:**

Nail-clipping and subungual debris of patients with above mentioned nail changes were subjected to KOH preparation. Culture was done on SDA and SDCCA media. Species identification was done by colony character, pigment production, LCB staining, and some special tests like germ tube test, etc.

**Results:**

Out of 85 cases, 44 cases showed the growth of fungus, amounting to 51.76% positivity. Among those 44 cases, the infective fungal agents were predominantly dermatophytes (50%), and the rest were due to yeasts (27.27%) and moulds (22.72%). Among the different species, *Trichophyton rubrum* (29.54%) accounted for the majority of dermatophytes; *Candida albicans* (11.78%) was the predominant yeast; and *Aspergillus niger* (18.18%) the commonest mold. No significant association could be established between the different fungal species and various clinical manifestations. Positive results were found more with fungal culture (95.45%) than KOH preparation (63.64%).

**Conclusion:**

The results show that nail changes are not always a reliable marker for predicting the causative organism, and relying only on the clinical manifestation (i.e., pattern of nail changes) in the diagnosis of onychomycosis is often misleading. The present study highlights the need for microbiological confirmation in case of onychomycosis.

## Introduction

Onychomycosis is one of the most common causes of deformed nails, and accounts for almost 50% of all nail diseases.^1^ Onychomycosis expresses itself in various forms, and the clinical types recognized by a recent classification scheme includes distal and lateral subungual onychomycosis, superficial onychomycosis, proximal subungual onychomycosis, endonyx onychomycosis and total dystrophic onychomycosis.^2^

Nail changes in onychomycosis can occur in various forms, viz. onychodystrophy, onycholysis, subungual hyperkeratosis, discoloration (melanonychia or leuconychia), or thickening of nail-plate. Not necessarily nail changes mentioned above should always be of fungal origin, since it has many clinical mimickers produced by other nail diseases, such as psoriasis, chronic onycholysis, lichen planus, chronic paronychia, hemorrhage or trauma, etc.^3^ So, it is advisable to obtain a definitive diagnosis of fungal infection before initiation of antifungal therapy in case of dystrophic nails.^4^ Mycological examination by direct microscopy (KOH preparation) combined with culture remains the gold standard technique, for its reasonable cost and least inconvenience to the patient.^5^

This study was undertaken to shed light on the clinicomycological correlation in fingernail onychomycosis with the aim of:

- Identifying the causative pathogens and demographic profile (age, sex and socioeconomic parameters) in patients attending urban hospital setting in eastern India.
- Determining the relationship between the clinical manifestations and causative agents.

## Materials and Methods

One hundred patients attending the dermatology OPD during the three-month period of the study with nail changes fitting to the inclusion criteria, which consisted of onychodystrophy, onycholysis, subungual hyperkeratosis, melanonychia, leuconychia and thickening of-nail plate were considered for the study. Fifteen patients who had received treatment either with topical and/or systemic antifungal agents for the present nail condition within the last one month were excluded. Thus, data from 85 patients were finally evaluated for the study. A thorough history was obtained and detailed clinical examination was performed for each of them regarding the dermatological conditions and some associated comorbidities. Relevant past medical records were also analyzed, while the patients were screened for the presence of diabetes and immunosuppression.

Nail clippings and subungual debris were collected and sent to the Microbiology Department for mycological study. Each sample was subjected to direct microscopy (KOH preparation) and culture was done on SDA and SDCCA for fungal isolation at 30°C for 3 weeks. The species were identified by colony character, pigment production, LCB staining, and some special tests like germ tube test, hair perforation tests etc.

For the purpose of the study, operational definition of onychomycosis was adopted as *any nails showing positive finding either with direct microscopy (KOH preparation) and/or fungal culture*. Statistical analysis was done using MedCalc^®^ version 7.0.0.2 software (http://www.medcalc.be) and *P*-value ≤0.05 was considered statistically significant.

## Results

Out of 85 cases studied, 44 cases showed the presence of fungus (either by KOH preparation and/or fungal culture) amounting to 51.76% positivity. Among those 44 cases, the mean age was 41.41 ± 14.64 years (range: 15-65 years) with a male-to-female ratio of 1:1. The patients mostly belonged to the upper middle-class (59%) background followed by lower middle-class (36.36%) and poor (4%) section of population. Occupation (especially wet work) was not found to have a significant association with onychomycosis in our study population. On comparing the comorbid conditions (diabetes and other endocrinopathies, hand eczema, immunosuppression), none of them were found significantly associated with onychomycosis (*P* -value >0.05); however, a small number of cases available for comparison may have influenced the results. Psoriasis was present in five cases showing fungal growth and four cases of non-onychomycosis with no significant association (*P* -value >0.05) (Table 1).

**Table 1:** Comparison of the comorbid conditions in the two sub-groups (onychomycosis and non-onychomycosis) of the study population (*N* = 85) showing nail changes

Comorbid condition	Onychomycosis	Non-onychomycosis	*P*-value*
Diabetes	3	3	0.74
Other endocrinopathies	4	5	0.91
Hand eczema	4	3	0.92
Immune suppression	4	1	0.4
Psoriasis	5	4	0.91

* On the basis of Chi-squared test with Yates correction and df = 1 for each condition

Infective fungal agents were predominantly dermatophytes (in 22 cases, i.e. 50%) and the rest were due to yeasts (in 12 cases, i.e., 27.27%) and molds (10 cases, i.e., 22.72%). Among the different species, *Trichophyton rubrum* (in 13 cases, i.e., 29.54%) amounted for the majority of dermatophytes; *Candida albicans* (in 10 cases, i.e., 22.72%) was the predominant yeast; and *Aspergillus niger* (in 8 cases, i.e., 18.18%) the commonest mold. In two cases (4.54%) of candidial infection, species identification was not possible by the laboratory methods available in our department (Table 2).

**Table 2:** The different categories of fungi isolated from fingernail onychomycosis with their comparative percentage of occurrence

Type of fungi	No of patients (*n* = 44)	Percentage of patients
Dermatophytes		
*Trichophyton rubrum*	13	29.54
*Trichophyton mentagrophytes*	6	13.63
*Trichophyton violaceum*	1	2.27
*Epidermophyton floccosum*	2	4.54
Total	22	50
Yeasts		
*Candida albicans*	10	22.72
Other *Candida*sp.	2	4.54
Total	12	27.27
Moulds		
*Aspergillus niger*	8	18.18
*Fusarium*sp.	2	4.54
Total	10	22.72

The association between clinical manifestations and onychomycosis was tested by Chi-square tests. It was found to have no significant association except for melanonychia (*P* = 0.0151) and subungual hyperkeratosis (*P* = 0.0299), which were found to be more significantly associated with non-onychomycotic etiology of nail changes. The sensitivity and specificity of the clinical manifestations were found to be very low (<90%) for them to be used as a diagnostic tool for determination of fungal origin of nail changes (Table 3).

**Table 3:** Association of the clinical manifestations of onychomycosis and their diagnostic value in the study population (*N* = 85)

Clinical manifestation	Number	Onychomycosis (*n* = 44)	Non-onychomycosis (*n* = 41)	Sensitivity	Specificity	*P*-value
Onychodystrophy	67	38	29	86.36	29.27	0.1344
Onycholysis	70	37	33	84.0	19.5	0.8802
Melanonychia	66	29	37	65.9	9.75	0.0151
Leuconychia	25	14	11	31.82	73.17	0.7901
Subungual hyperkeratosis	66	23	43	52.27	73.17	0.0299
Thickening	15	9	6	20.45	85.36	0.6755
Paronychia	42	21	21	47.73	48.78	0.9166

No significant association was found between the different species of fungus and various clinical manifestations when the factors were subjected to univariate analysis. Result shows that clinical manifestations (i.e., pattern of nail changes) are distributed in such a way that there exists no species specificity (Table 4).

**Table 4:** Association of the clinical manifestations of onychomycosis (*N* = 44) with their respective etiological agent (different species of fungi)

Clinical manifestation	Number	*Trichophyton rubrum* (*n* = 13)	*Candida albicans* (*n* = 10)	*Aspergillus niger* (*n* = 8)	Others* (*n* = 13)
Onychodystrophy	67	10 (0.85)	8 (0.75)	8 (0.28)	7 (0.04)
Onycholysis	70	9 (0.34)	10 (0.26)	6 (0.93)	8 (0.08)
Melanonychia	66	7 (0.06)	6 (0.31)	8 (0.25)	11 (0.77)
Leuconychia	25	7 (0.08)	4 (0.68)	2 (0.9)	5 (0.65)
Subungual hyperkeratosis	66	7 (0.06)	4 (0.73)	4 (0.82)	6 (0.009)
Thickening	15	3 (0.87)	2 (0.81)	2 (0.93)	4 (0.34)
Paronychia	42	8 (0.51)	2 (0.1)	8 (0.008)	5 (0.57)

Figures in the parentheses indicate *P*-value calculated on the basis of χ^2^ test (Yates correction was applied) comparing clinical manifestation in specific species and rest of the persons.

* This group includes other *Trichophyton* species, *Candida non-albicans*, and *Fusarium* species

Direct microscopy (KOH preparation) was positive in 28 (63.64%) cases and fungal culture in 42 (95.45%) cases out of the 44 cases diagnosed with onychomycosis. False negative result was found in 16 (36.36%) cases with direct microscopy (KOH preparation) and in 2 (4.54%) cases with fungal culture (Table 5).

**Table 5:** KOH preparation (direct microscopy) vs. fungal culture in the diagnosis of onychomycosis (*N* = 85)

Diagnostic tests	Fungal culture	Fungal culture	Total
	positive	negative
KOH positive	26	2	28
KOH negative	16	41	57
Total	42	43	85

## Discussion

The present study confirmed once again that diagnosis of onychomycosis relying only on the pattern of nail changes is often misleading since only 51.76% of the cases showing nail changes mimicking onychomycosis showed the presence of fungus. Other workers from different geographical locations had reported similar results with the incidence of fungal etiology in patients with nail changes clinically pre-diagnosed as onychomycosis in only 43.7% in Poland,^4^50.6% in Turkey,^6^ and 45.53% in India.^7^ We have found onychomycosis to be a disease of the middle-aged, with males and females being equally susceptible in our hospital setup. A study from central India found males to be the primary sufferer (male-to-female ratio of 3:1),^8^ whereas females were reported to be primarily affected (in 96% cases) in another study from Bangalore, India.^7^ But, both the studies revealed onychomycosis to be a disease of the middle-aged (mean age 29.4 years;^8^ age range between 21 and 40 years^7^).

Our study has documented dermatophytes as the primary pathogen involved, whereas *T. rubrum* (present in 29.54% cases) remains the most common fungus responsible for onychomycosis. Studies from across the globe^9^–^12^ as well as in India^8^,^13^,^14^ found the same predominant etiology, which proves by itself that *T. rubrum* still remains the most common etiological agent in producing onychomycosis. A study on a group of immunocompromised patients suffering from SLE showed *T. rubrum* as the principal agent for onychomycosis,^15^ although another study on autoimmune subject with onychomycosis found *Candida* spp. to be more frequent as compared to dermatophytes.^16^ A few recent reports have found *Candida* spp.^7^,^17^ and *Aspergillus niger*^18^ to be the predominant agents, which can be because of changed microbial milieu in their environment or contamination, respectively. Our study found *Candida albicans* in 22.72% cases and *Aspergillus niger* in 18.18% cases, accounting for next two commonest pathogens after *T. rubrum*, suggesting that they are gradually emerging as important etiological agents in onychomycosis. Since nothing can predict change in the microbiological environment, and the fact that therapy is directed by the type of the organism, it becomes imperative that this kind of studies should be performed at regular intervals to find out any change in the etiological agent.

In our attempt in finding a diagnostic clue to predict fungal involvement in patients with deformed nails, we observed melanonychia (*P* = 0.01) and subungual hyperkeratosis (*P* = 0.03) to have significant association with non-onychomycotic nail changes, suggesting that in these events we need to be more cautious in attributing these changes to fungal etiology. While *Candida* and/or *Aspergillus* were isolated predominantly in a previously reported study on fungal melanonychia,^19^ we could not find any such species-specific association. Paronychia has been reported to be more commonly associated with *Candida*^10^ and non-dermatophytic onychomycosis.^20^ However, our study did not reveal any statistically significant association between any particular species of fungus and the clinical features we analyzed.

While analyzing the results of direct microscopy (KOH preparation) in detecting onychomycosis, we concluded that, if we depended solely upon the results of KOH preparation, we would have missed a good number (16 cases, i.e., 36.36%) of patients, and so we fully agree with Feuilhade de Chauvin M^21^ in concluding that direct microscopy must always be coupled with fungal culture for accurate diagnosis and allowing correct species identification. Similar low positive results with direct microscopy (KOH preparation) were also found by other workers,^22^ that is, 73.8% sensitivity for dermatophytes and 67.2% sensitivity for proven etiologic non-dermatophytic fungus. Presently, new methods are being tried to improve the diagnostic yield, which include sample collection by microdrilling,^23^ polymerase chain reaction,^24^ and histopathological examination of nails.^25^ These methods if proved cost-effective can further help in diagnosing onychomycosis.

## Conclusion

The present study highlights the need for microbiological confirmation in case of onychomycosis and stresses that the type of nail changes cannot be taken as a reliable marker for predicting the causative organism. The study has also elaborated the prevalence of different fungi causing fingernail onychomycosis in an urban setting in the eastern part of India, which can provide useful guidelines for the appropriate management of cases and further epidemiological study.
